# Simple Equations Method and Non-Linear Differential Equations with Non-Polynomial Non-Linearity

**DOI:** 10.3390/e23121624

**Published:** 2021-12-02

**Authors:** Nikolay K. Vitanov, Zlatinka I. Dimitrova

**Affiliations:** 1 Institute of Mechanics, Bulgarian Academy of Sciences, Acad. G. Bonchev Str., Block 4, 1113 Sofia, Bulgaria; zdim@imbm.bas.bg; 2 Climate, Atmosphere and Water Research Institute, Bulgarian Academy of Sciences, Blvd. Tzarigradsko Chaussee 66, 1784 Sofia, Bulgaria

**Keywords:** non-linear differential equations, exact solutions, simple equations method (SEsM), composite functions, Faa di Bruno formula, V-function

## Abstract

We discuss the application of the Simple Equations Method (SEsM) for obtaining exact solutions of non-linear differential equations to several cases of equations containing non-polynomial non-linearity. The main idea of the study is to use an appropriate transformation at Step (1.) of SEsM. This transformation has to convert the non-polynomial non- linearity to polynomial non-linearity. Then, an appropriate solution is constructed. This solution is a composite function of solutions of more simple equations. The application of the solution reduces the differential equation to a system of non-linear algebraic equations. We list 10 possible appropriate transformations. Two examples for the application of the methodology are presented. In the first example, we obtain kink and anti- kink solutions of the solved equation. The second example illustrates another point of the study. The point is as follows. In some cases, the simple equations used in SEsM do not have solutions expressed by elementary functions or by the frequently used special functions. In such cases, we can use a special function, which is the solution of an appropriate ordinary differential equation, containing polynomial non-linearity. Specific cases of the use of this function are presented in the second example.

## 1. Introduction

Complex systems are numerous in human societies and in Nature. Several examples are research groups, communities, traffic networks, stock markets, etc. [1,2,3,4,5,6,7,8]. The above (and many other) complex systems are important. Because of this, their dynamics are studied by many research groups [9,10,11,12,13,14,15,16,17,18,19,20,21,22,23]. Characteristic feature of the most complex systems is their non-linearity. Examples for this can be seen in fluid mechanics, solid state physics, etc. [24,25,26,27,28,29,30,31]. In many cases, the non-linearity is modeled by non-linear differential or difference equations [32,33,34,35,36,37,38,39,40,41,42,43,44,45,46,47,48,49]. Thus, the study of the analytical and numerical solutions of the non-linear differential equations are of large interest for science and for practice. We note that the exact solutions of nonlinear differential equations are of interest also for the numerical analysis of these equations. The reason is that the exact solutions can be used to test the corresponding numerical methods and computer programs.

The research on the exact solutions of the non-linear differential equation started a long time ago. At the beginning, the efforts were directed at removing the non-linearity of the solved equation by means of an appropriate transformation. A large success in this direction was the Hopf–Cole transformation [50,51]. It transforms the non-linear Burgers equation to the linear heat equation. An even larger success was the transformation which connected the Korteweg–de Vries equation to the famous linear equation of Schrödinger. This transformation leaded to the development of t the Method of Inverse Scattering Transform [52,53,54]. Another method which uses an appropriate transformation is the method of Hirota [55,56]. Truncated Painleve expansions lead to many of these appropriate transformations [57,58,59,60,61].

Below, we discuss the SEsM (*Simple Equations Method*) for obtaining exact solutions of non-linear differential equations. Before the arising of SEsM, Kudryashov formulated the Method of Simplest Equation (MSE) [62]. MSE is based on the determination of the singularity order *n* of the solved NPDE and on searching of a particular solution of this equation as a series containing powers of solutions of a simpler equation called the simplest equation [63,64,65,66]). Kudryashov [67,68,69,70] also used various transformations in order to transform the non-linearity of a generalized evolution equation of the wave dynamics and to obtain exact solutions of this equation. More results connected to the application of the MSE can be seen, for example, in [71,72,73,74,75,76,77].

Some elements of SEsM [77,78,79,80,81] can be seen in our publications written years ago [82,83,84,85,86,87,88]. About 10 years ago [89,90], we used the ordinary differential equation of Bernoulli as the simplest equation [91] and applied a methodology called the Modified Method of Simplest Equation (MMSE) to model non-linear partial differential equations from ecology and population dynamics [92]. We have used the concept of the balance equation, which helps us to determine the kind of the simplest equations in the MMSE. The balance equation also helps us to determine the form of the solution of the solved equations as a series of the solution of the simplest equation [93,94]. The methodology based on the truncation of the series of solutions of the simplest equation by means of application of a balance equation is equivalent to the MSE mentioned above. We made many applications of MMSE for obtaining exact solutions of different non-linear differential equations [95,96,97,98,99,100,101,102,103]. We note the article [102] which is connected to the part of the topics discussed below in the text.

Over the course of the years, MMSE was extended to SEsM [77]. SEsM is connected to the possibility of using more than one simple equation in order to construct the solution of the solved non-linear differential equation. A realization of this possibility was shown in [104]. The first description of SEsM was made in [78] and then in [77,79,80,81,105]. Several examples of the methodology and its applications are presented in [106,107,108,109,110,111]. An important part of SEsM is the construction of the solution of the solved equation. This solution is a composite function of solutions of more simple differential equations. Thus, the Faa di Bruno formula for the derivative of the composite function participates in the process of obtaining exact solutions [112,113]. The possibility for use of more than one simple equation and the possibility of use complicated composite functions which connect the solution of the solved equation and the solutions of the simple equation distinguish SEsM from the methodology of Kudryashov and from other methodologies such as the Modified Simple Equation Method [114,115]. We note also that SEsM can be easily connected to other approaches (for examples, see [116,117,118]).

Up to now, SEsM can deal successfully with many non-linear differential equations which contain polynomial non-linearity. In this article, we start to study the topic of the application of SEsM to non-linear differential equations with non-polynomial non-linearity. Our approach will be similar to many of the approaches described above. We will search for appropriate transformations. These transformations will transform the non-polynomial non-linearity of the solved equations to polynomial non-linearity. After this, we will use the capacity of the SEsM to obtain exact analytical solutions of many equations containing polynomial non-linearity.

The text below is organized as follows. We describe the SEsM in Section 2. We note that the general case of the SEsM is for a system of non-linear differential equations. Below, we consider the specific case of search for exact traveling wave solutions of one non-linear differential equation. In Section 3, we discuss the properties of the appropriate transformations and formulate two propositions about these properties. Several appropriate transformations are listed. In Section 4, we present two selected examples of application of SEsM to non-linear differential equations with non-polynomial non-linearity. The first example illustrates the reduction of the solved non-linear differential equation to a system of non-linear algebraic equations containing relationships between the parameters of the solved equation and the parameters of the constructed solution. The second example illustrates the fact that there are simple equations in the SEsM for which solutions cannot be constructed by elementary functions or by the known special functions. Because of this, we emphasize the usefulness of a special function, which is a solution of an ordinary differential equation containing polynomial non-linearity. This function plays important role in the construction of many solutions of non-linear differential equations by means of SEsM. Several concluding remarks are summarized in Section 5. The text ends with two appendices. Appendix A gives the necessary information about the derivatives of the composite functions. Appendix B supplies useful information connected to the use of composite functions and their derivatives in SEsM.

## 2. The Simple Equations Method (SEsM)

**Figure 1:**
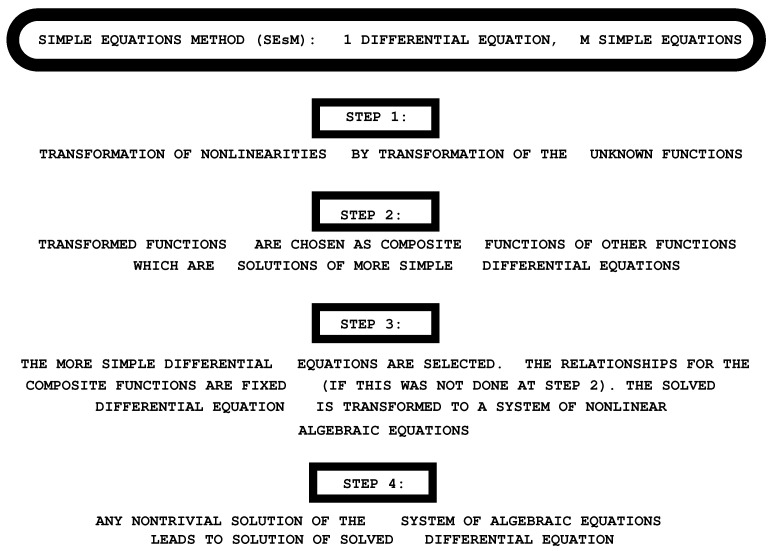
The Simple Equations Method (SEsM) for the specific case of one solved equation by use of *M* simple equations. The method has four steps which are described in the text. The discussion in the text below is about the kinds of possible transformations used in Step (1.) of SEsM.

In general, the SEsM is designed for obtaining exact solutions of systems of differential equations. Below, we are going to discuss the specific case when one wants to obtain exact solutions of a single nonlinear differential equation. The summary of this specific case of SEsM is as follows—Figure 1. We consider the (partial or ordinary) differential equation:

(1) Y[u(x,⋯,t),⋯)]=0.

In (1), Y[u(x,⋯,t),⋯] depends on the function u(x,⋯,t) and some of its derivatives (*u* can be a function of several spatial coordinates). In order to obtain an exact solution of (1), we make the following four steps.

**Step** **(1.)** **Transformation of the non-linearity of the solved equation**
  We apply the following transformations:
  (2) $$u\left(x,\ldots ,t\right)=T[F_{1}\left(x,\cdots ,t\right),\cdots ,F_{N}\left(x,\cdots ,t\right)].$$
  T(F) is a function of another functions $F_{i}$, i=1,⋯,N. $F_{i}\left(x,\cdots ,t\right)$ is a function of several spatial variables as well as of the time. The transformation *T* may remove some non-linearity if possible. An example of such a transformation is the Hopf–Cole transformation which leads to the linearization of the Burgers equation [50,51]). Moreover, *T* may transform the non-linearity of the solved differential equations to a more treatable kind of non-linearity. Transformations with this property will be discussed below in the text.
  In numerous cases, one may skip this step (then we have u(x,…,t)=F(x,…,t)). In many other cases, the transformation is needed for obtaining a solution of the studied non-linear PDE. The application of (2) to (1) leads to non-linear differential equations for the functions $F_{i}$. We do not know the general form for the transformation *T*. The reason is that the non-linearity in the solved equations can be of different kinds.
  We note that Step (1.) of SEsM will be at the focus of our study in this article. We are going to study non-linear equations for which non-linearity can be reduced to polynomial non-linearity by means of appropriate transformations.
**Step** **(2.)** **The solution is searched as composite function of solutions of more simple equations**
  In this step, the functions $F_{i}\left(x,\ldots ,t\right)$ are chosen as composite functions of functions $f_{1},\ldots$, which are solutions of more simple differential equations. In general, we do not fix the relationship for the composite function. Then, we use the general Faa di Bruno relationship for the derivatives of a composite function [113]. In MMSE, we have used a fixed relationship for the composite function. For an example, for the case of 1 solved equation and one function *F*:
  (3) $$\begin{array}{c} F=\alpha +\sum_{i_{1}=1}^{N}\beta_{i_{1}}f_{i_{1}}+\sum_{i_{1}=1}^{N}\sum_{i_{2}=1}^{N}\gamma_{i_{1},i_{2}}f_{i_{1}}f_{i_{2}}+\sum_{i_{1}=1}^{N}\cdots \sum_{i_{N}=1}^{N}\sigma_{i_{1},\cdots ,i_{N}}f_{i_{1}}\cdots f_{i_{N}}. \end{array}$$
**Step** **(3.)** **Selection of the simple equations**
  We select the simple equations for the functions $f_{1},\ldots$. In addition, we have to fix the relationship between the composite functions $F_{i}\left(x,\ldots ,t\right)$ and the functions $f_{1},\ldots$. We note that the fixation of the simple equations and the fixation of the relationships for the composite functions are connected. The reason for this is as follows. The fixations transform the left-hand sides of the solved equations. The result of this transformation can be functions which are sums of terms. Each term contains some function multiplied by a coefficient. The coefficient is a relationship connecting some of the parameters of the solved equations and some of the parameters of the solutions of the used simple equations. Each coefficient must have at least two terms (Otherwise, the trivial solution will be produced). In order to ensure this, a balance procedure must be applied. This balance procedure leads to one or more additional relationships among the parameters of the solved equation and parameters of the solutions of the used simple equations. The additional relationships are called balance equations. The balance equations are the connection between the choice of the simple equation and the fixation of the form of the composite function.
**Step** **(4.)** **Solution of the obtained system of non-linear algebraic equations**
  We may obtain a nontrivial solution of (1) if all coefficients mentioned in Step (3.) are set to 0. This condition leads to a system of non-linear algebraic equations. The equations connect the coefficients of the solved non-linear differential equation and for the coefficients of the solutions of the simple equations. Any nontrivial solution of this algebraic system leads to a solution of the studied non-linear partial differential equation.
  There are two possibilities for the solution of the system of non-linear algebraic equations:
  - The number which is the sum of the number of parameters of the solution and the number of parameters of the equation can be larger than the number of algebraic equations or equal to the number of algebraic equations. Then, the system usually (but not in all of the cases) has a nontrivial solution(s). Independent parameters may be presented in this situation. The other parameters of the solution are functions of these independent parameters.
  - The number which is the sum of the number of parameters of the solution and the number of parameters of the equation is smaller than the number of algebraic equations. Then, the system of algebraic equations usually does not have a nontrivial solution. However, there can be important exceptions to this. An exception occurs when the number of equations of the algebraic system can be reduced and this number becomes less or equal to the number of available parameters. Then, this case is reduced to the previous one and a nontrivial solution is possible.

## 3. Several Transformations Which Are of Interest for the SEsM

### 3.1. General Considerations

Below, we discuss in more detail the application of Step (1.) of the SEsM to differential equations containing non-polynomial non-linearity. The idea is to reduce the non-polynomial non-linearity to polynomial non-linearity and then to deal with the polynomial non-linearity by means of Steps (2.), (3.), and (4.) of the SEsM.

We consider below the problem of searching for exact solutions of non-linear differential equations containing the function u(x,…,t) and its derivatives. In general, we consider the case of several spatial variables x,… and the time *t*.

**Proposition** **1.** *Let us consider a differential equation for the function u(x,…,t) which contains terms of two kinds:*

1. *Terms containing only derivatives of u;*
2. *Terms containing one or several non-polynomial non-linearities of the function u and these non-polynomial non-linearity are of the same kind.*

*Let u=T(F) be a transformation with the following properties:*

1. *Property 1: The transformation T transforms any of the non-polynomial non-linearity to a function which contains only polynomials of F.*
2. *Property 2: The transformation T transforms the derivatives of u to terms containing only polynomials of derivatives of F or polynomials of derivatives of F multiplied or divided by polynomials of F.*

*Then, the transformation T transforms the studied differential equation to a differential equation containing only polynomial non-linearity of F.*

**Proof.**  The studied differential equation contains two kinds of terms: derivatives of *u* and terms which contain the non-polynomial non-linearity of *u*. These non-polynomial non-linearities are of the same kind. Let us apply the transformation u=T(F) to the terms of our differential equation. We consider first each of the terms containing derivatives. According to Property 2 of the transformation *T*, it transforms this term to a term containing derivatives of *F* and polynomials of *F*. What remains are the terms containing the non-polynomial non-linearity. Because of the property 1 of the transformation *T*, each of these terms are transformed to a term containing only polynomials of *F*. Then, the transformation *T* transforms the studied differential equation for *u* to a differential equation containing only polynomials of *F* as well as derivatives of *F*. □

### 3.2. Several Kinds of Non-Linearity Possessing the Properties 1 and 2 from the Proposition above

Let us now consider several transformations which have the properties discussed in the above Proposition. We note that there are more transformations of the desired kind than the transformations listed below, and one of them will be used in the Example 2 in the next Section.

**Case** **1:** N(u)=exp(u); $N\left(u\right)={\left[\exp\left(u\right)\right]}^{m}$
  In this case, the transformation is u=ln(F). Let us consider first the case N(u)=exp(u). The transformation has Property 1 as follows:
  N(u)=exp(u)=exp[ln(F)]=F.
  The transformation has also Property 2. For an example:
  $$u_{x}={\left[\ln\left(F\right)\right]}_{x}=\frac{F_{x}}{F}.$$
  The derivative of *u* contains only a derivative of *F* in the nominator and a polynomial of *F* in the denominator. We note that further differentiation does not change the situation. For an example:
  $$u_{xx}=\frac{F_{xx}}{F}-\frac{F_{x}^{2}}{F^{2}};\;\;\;u_{xt}=\frac{F_{xt}}{F}-\frac{F_{x}F_{t}}{F^{2}}.$$
  Next, we consider the case $N\left(u\right)={\left[\exp\left(u\right)\right]}^{m}$. In this case, $N\left(u\right)={[\exp[\left(\ln\left(F\right)\right]}^{m}=F^{m}$. The transformation has Property 1. In addition, the transformation that has Property 2 as the relationship u=ln(F) also holds. Thus, the transformation works also for the non-linearity of the kind ${\left[N\left(u\right)=\exp\left(u\right)\right]}^{n}$.
**Case** **2:** N(u)=sin(u); $N\left(u\right)={\left[\sin\left(u\right)\right]}^{m}$.
  In this case, a possible transformation is $u=4\tan^{-1}\left(F\right)$. Let us consider first the case N(u)=sin(u). The transformation has Property 1 as follows:
  $$N\left(u\right)=\sin\left[4\tan^{-1}\left(F\right)\right]=4\frac{F(1-F^{2})}{{\left(1+F^{2}\right)}^{2}}$$
  N(u) is transformed to a function which contains only polynomials of *F*. The transformation also has Property 2, for an example:
  $$u_{x}=\frac{4}{1+F^{2}}F_{x}$$
  Next, we consider the case $N\left(u\right)={\left[\sin\left(u\right)\right]}^{m}$. In this case, the transformation has Property 1 as follows:
  $$N\left(u\right)={\left\{\sin\left[4\tan^{-1}\left(F\right)\right]\right\}}^{m}=4^{m}\frac{{\left[F\left(1-F^{2}\right)\right]}^{m}}{{\left[{\left(1+F^{2}\right)}^{2}\right]}^{m}}.$$
  The last relationship shows that N(u) is transformed to a function containing only polynomials of *F*. Since u=4tan−1(F), Property 2 holds.
**Case** **3:** N(u)=cos(u); $N\left(u\right)={\left[\cos\left(u\right)\right]}^{m}$.
  We consider first the case N(u)=cos(u). The transformation is $u=4\tan^{-1}\left(F\right)$. The transformation has Property 1 as follows:
  $$N\left(u\right)=\frac{{\left(1-F^{2}\right)}^{2}-4F^{2}}{{\left(1+F^{2}\right)}^{2}}$$
  The transformation also has Property 2 as follows:
  $$u_{x}=\frac{4}{1+F^{2}}F_{x}.$$
  For the case $N\left(u\right)={\left[\cos\left(u\right)\right]}^{m}$, the transformation has Property 1 as follows:
  $$N\left(u\right)=\frac{{\left[{\left(1-F^{2}\right)}^{2}-4F^{2}\right]}^{m}}{{\left[{\left(1+F^{2}\right)}^{2}\right]}^{m}}$$
  The transformation also has Property 2.
**Case** **4:** N(u)=tan(u); $N\left(u\right)={\left[\tan\left(u\right)\right]}^{m}$.
  We first consider the case N(u)=tan(u). In this case, a possible transformation is $u=\tan^{-1}\left(F\right)$. The non-linearity is transformed to a polynomial of *F*: $N\left(u\right)=\tan[\tan^{-1}\left(F\right)]=F$. The derivative of *u* is also reduced to a relationship containing the polynomial of *F* and a derivative of *F*. For an example:
  $$u_{x}=\frac{1}{1+F^{2}}F_{x}.$$
  For the case $N\left(u\right)={\left[\tan\left(u\right)\right]}^{m}$, N(u) is transformed to the polynomial $F^{m}$. The derivative of *u* remains as above.
**Case** **5:** N(u)=cot(u); $N\left(u\right)={\left[\cot\left(u\right)\right]}^{m}$.
  The transformation in this case is $u=\cot^{-1}\left(F\right)$. The non-linearity is transformed to a polynomial of *F*: $N\left(u\right)=\cot[\cot^{-1}\left(F\right)]=F$. The derivative of *u* is reduced to a relationship containing a polynomial of *F* and a derivative of F. For an example:
  $$u_{x}=-\frac{1}{1+F^{2}}F_{x}$$
  For the case $N\left(u\right)={\left[\cot\left(u\right)\right]}^{m}$, N(u) is transformed to the polynomial $F^{m}$. The derivative of *u* remains as above.
**Case** **6:** N(u)=sinh(u); $N\left(u\right)={\left[\sinh\left(u\right)\right]}^{m}$.
  In this case, the transformation is $u=4\tanh^{-1}\left(F\right)$. The derivatives of *u* contain derivatives of *F* and polynomials of *F*. For an example:
  $$u_{x}=\frac{4}{1-F^{2}}F_{x}$$
  The non-linearity is reduced as a relationship containing polynomial non-linearity of *F*. For the case N(u)=sinh(u) we obtain:
  $$N\left(u\right)=\sinh\left[4\tanh^{-1}\left(F\right)\right]=4\frac{F(1+F^{2})}{{\left(1-F^{2}\right)}^{2}}$$
  For the case $N\left(u\right)={\left[\sinh\left(u\right)\right]}^{m}$ we obtain:
  $$N\left(u\right)=4^{m}\frac{{\left[F\left(1+F^{2}\right)\right]}^{m}}{{\left[{\left(1-F^{2}\right)}^{2}\right]}^{m}}$$
**Case** **7:** N(u)=cosh(u); $N\left(u\right)={\left[\cosh\left(u\right)\right]}^{m}$.
  In this case, the transformation is $u=4\tanh^{-1}\left(F\right)$. For the case N(u)=cosh(u):
  $$N\left(u\right)=\frac{{\left(1+F^{2}\right)}^{2}+4F^{2}}{{\left(1-F^{2}\right)}^{2}}$$
  The transformation also has Property 2, for an example:
  $$u_{x}=\frac{4}{1-F^{2}}F_{x}$$
  For the case $N\left(u\right)={\left[\cosh\left(u\right)\right]}^{m}$, the transformation has the Property 1 as follows:
  $$N\left(u\right)=\frac{{\left[{\left(1+F^{2}\right)}^{2}+4F^{2}\right]}^{m}}{{\left[{\left(1-F^{2}\right)}^{2}\right]}^{m}}$$
**Case** **8:** N(u)=tanh(u); $N\left(u\right)={\left[\tanh\left(u\right)\right]}^{m}$.
  In this case, the transformation is $u\left(F\right)=\tanh^{-1}\left(F\right)$. N(u) is reduced to N(u)=F, which is a polynomial of *F*. The derivatives of *u* contains polynomials of *F* and derivatives of *F*. For an example:
  $$u_{x}=\frac{1}{1-F^{2}}$$
**Case** **9:** N(u)=coth(u); $N\left(u\right)={\left[\coth\left(u\right)\right]}^{m}$.
  In this case, the transformation is $u\left(F\right)=\coth^{-1}\left(F\right)$. N(u) is reduced to N(u)=F, which is a polynomial of *F*. The derivatives of *u* contains polynomials of *F* and derivatives of *F*. For an example:
  $$u_{x}=-\frac{1}{F^{2}-1}$$
**Case** **10:** N(u)=sin(mu); N(u)=cos(mu).
  In this case, we can use the following relationships:
  (4) sin(mu)=∑k=0mmkcosk(u)sinm−k(u)sinπ2(m−k)
  (5) cos(mu)=∑k=0mmkcosk(u)sinm−k(u)cosπ2(m−k)
  The transformation is $u=4\tan^{-1}F$. The case N(u)=sin(mu) has Property 1 as follows:
  N(u)=∑k=0mmkcosk(u)sinm−k(u)sinπ2(m−k)=∑k=0mmk(1−F2)2−4F2(1+F2)2k4F(1−F2)(1+F2)2m−ksinπ2(m−k).
  As above, the transformation has Property 2. For the case N(u)=cos(mu):
  N(u)=∑k=0mmkcosk(u)sinm−k(u)sinπ2(m−k)=∑k=0mmk(1−F2)2−4F2(1+F2)2k4F(1−F2)(1+F2)2m−kcosπ2(m−k).

The list of the appropriate transformations can be continued. Let us now consider two examples.

## 4. Two Illustrative Examples

### 4.1. Example 1

We consider the following equation:

(6) $$bu_{xx}^{2}+du_{tt}^{2}=l\sin^{2}\left(u\right)$$

where b,d,andl are parameters. Following the considerations from the previous section, we use the transformation $u=4\tan^{-1}\left(F\right)$ at the first step of the application of the SEsM. This transformation leads to the following equation for F(x,t), containing only polynomial non-linearities:

(7) $$\begin{array}{c} 4F^{2}\left(bF_{x}^{4}+dF_{t}^{4}\right)-4\left(F+F^{3}\right)\left(bF_{x}^{2}F_{xx}+dF_{t}^{2}F_{tt}\right)+\left(1+2F^{2}\right)\left(bF_{xx}^{2}+dF_{tt}^{2}\right)+F^{4}\left(bF_{xx}+dF_{tt}\right)-l\left(F^{6}-2F^{4}+F^{2}\right)=0 \end{array}$$

Step (2.) of the SEsM requires *F* to be a composite function of more simple functions: $F\left(x,t\right)=F[T_{1}\left(x,t\right),T_{2}\left(x,t\right)]$. In order to consider the general case, we have to use the information from Appendices 1 and 2. In order to keep the example relatively simple, we will consider a particular case of the above composite function:

(8) $$F\left(x,t\right)=AT_{1}\left(\mu \right)T_{2}\left(\xi \right),$$

where μ=αx and ξ=γt. This means that we are going to search for standing wave solutions of (6). (8) leads to large simplifications of the corresponding Faa di Bruno formulas. The result is a differential equation which contains polynomials constructed of $T_{1}$, $T_{2}$, and their derivatives.

At Step (3.) of the SEsM, we have to determine the form of the functions $T_{1}$ and $T_{2}$. Following the methodology of SEsM, we assume that $T_{1}$ and $T_{2}$ are solutions of more simple (and ordinary) differential equations which contain polynomial non-linearity.

(9) $$\begin{array}{c} {T_{1}}_{\mu }^{2}=\sum_{i=0}^{N_{1}}\delta_{i}T_{1}^{i};\\ {T_{2}}_{\xi }^{2}=\sum_{j=0}^{N_{2}}\epsilon_{i}T_{2}^{j}, \end{array}$$

where $\delta_{i}$ and $\epsilon_{i}$ are parameters. The simple equations are ordinary differential equations containing polynomial non-linearity. These equations are particular cases of the Equation (A7).

We substitute (9) in the relationship which occurs in Step (2.) of the SEsM. As a result, we obtain a polynomial of $T_{1}$ and $T_{2}$, which contains monomials of $T_{1}$ and $T_{2}$ and monomials which are combinations of powers of $T_{1}$ and $T_{2}$. These monomials are multiplied by coefficients which are non-linear algebraic relationships containing the parameters of the solved equation and the parameters of the more simple equations (9). We have to ensure that any of these non-linear algebraic relationships contains at least two terms. This is performed by a balance procedure which leads to the fixation of the values of the parameters $N_{1}$ and $N_{2}$. Below, we consider a specific case: the case when $N_{1}=N_{2}=4$. For this case:

(10) $$\begin{array}{c} {T_{1}}_{\mu }^{2}=\delta_{4}T_{1}^{4}+\delta_{3}T_{1}^{3}+\delta_{2}T_{1}^{2}+\delta_{1}T_{1}+\delta_{0};\\ {T_{2}}_{\xi }^{2}=\epsilon_{4}T_{2}^{4}+\epsilon_{3}T_{2}^{3}+\epsilon_{2}T_{2}^{2}+\epsilon_{1}T_{1}+\epsilon_{0}. \end{array}$$

In order to keep the example simple, we further restrict the form of the simple equations by setting $\delta_{3}=\delta_{1}=\epsilon_{3}=\epsilon_{1}=0$, and in addition, we assume $\delta_{4}=p,\delta_{2}=q,and\;\delta_{0}=r$ as well as $\epsilon_{4}=s,\epsilon_{2}=v,and\;\epsilon_{0}=w$. In such a way, the simple equations for the function $T_{1}$ and $T_{2}$ become:

(11) $$\begin{array}{c} {T_{1}}_{\mu }^{2}=pT_{1}^{4}+qT_{1}^{2}+r;\\ {T_{2}}_{\xi }^{2}=sT_{2}^{4}+vT_{2}^{2}+w. \end{array}$$

The form (11) of the simple equations lead to the following system of non-linear algebraic equations (these are the non-linear algebraic relationships for the coefficients of the polynomial containing $T_{1}$, $T_{2}$, and their derivatives:

(12) $$\begin{array}{ccc} b\alpha^{4}q^{2}+d\gamma^{4}v^{2} & = & l\\ d\gamma^{4}vA^{2}w-b\alpha^{4}pq & = & 0\\ -4d\gamma^{4}sw-b\alpha^{4}q^{2}-4b\alpha^{4}pr+l-d\gamma^{4}v^{2} & = & 0\\ b\alpha^{4}A^{4}r^{2}+d\gamma^{4}s^{2} & = & 0\\ -b\alpha^{4}qA^{2}r+d\gamma^{4}sv & = & 0\\ b\alpha^{4}p^{2}+d\gamma^{4}A^{4}w^{2} & = & 0\\ -d\gamma^{4}sv+b\alpha^{4}qA^{2}r & = & 0\\ -d\gamma^{4}vA^{2}w+b\alpha^{4}pq & = & 0 \end{array}$$

The system (12) has the following solution:

(13) $$p=w=r=s=0,\;\;q=\delta \frac{{\left[-b\left(d\gamma^{2}v^{2}-l\right)\right]}^{1/2}}{\alpha^{2}b},\;\;\delta =\pm 1,\;\;-b\left(d\gamma^{2}v^{2}-l\right)\geq 0$$

and *v*, *A*, *l*, *b*, *d*, α, γ are free parameters (they have to satisfy the condition $-b(d\gamma^{2}v^{2}-l)\geq 0$. Equation (13) corresponds to the following solution of (6):

(14) $$u\left(x,t\right)=4\tan^{-1}\left\{A\exp\left[\delta_{1}\left(\alpha vx+\gamma \delta \frac{{\left[-b\left(d\gamma^{2}v^{2}-l\right)\right]}^{1/2}}{\alpha^{2}b}t\right)\right]\right\},\;\;\delta_{1}=\pm 1.$$

**Figure 2:**
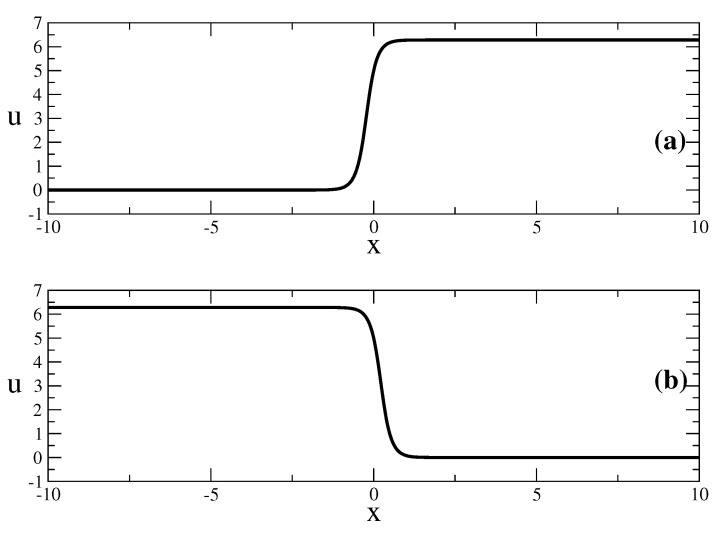
Examples of a kink and anti-kink described by the solution (14). The parameters are as follows: t=0.2. v=0.2, A=3, l=1.3, b=1, d=2.2α=0.08, γ=2.1, δ=1.0d0. $\delta_{1}=1$ for (**a**); $\delta_{1}=-1$ for (**b**).

This solution describes traveling waves of kind kink and anti-kink—Figure 2

### 4.2. Example 2

By this example, we are going to illustrate the use of a transformation which transforms non- polynomial non-linearity to polynomial non-linearity and is not listed in Section 3. In addition, we illustrate the fact that in many cases, the solutions of the more simple equations cannot be composed by elementary functions. Because of this, we will need an appropriate special function. Such a function will be discussed below.

Let us consider the following equation:

(15) $$au_{xx}-bu_{tt}=c\sinh\left(u\right)+\frac{1}{3}d\sinh\left(\frac{u}{3}\right)+\frac{2}{3}e\sinh\left(\frac{2u}{3}\right),$$

where a,b,c,d,e are parameters. In order to keep the calculations simple, we will consider the case of traveling waves with the corresponding coordinate ξ=αx+βt. At Step (1.) of the SEsM, we apply the following transformation:

(16) $$u\left(x,t\right)=A\cosh^{-1}\left(F\right),\;\;A=3.$$

The transformation (16) leads to the following equation for *F*:

(17) $$\frac{9}{2}\left(\alpha^{2}a-\beta^{2}b\right)F_{\xi }^{2}=4cF^{5}+2eF^{4}+\left(d-7c\right)F^{3}-3eF^{2}-\left(d-3c\right)F+e$$

Next, we have to construct *F* by functions which are solutions of more simple differential equations. We note the following. In [102], we mentioned a function which is the solution of the following equation with polynomial non-linearity:

(18) $${\left(\frac{d^{k}g}{d\xi^{k}}\right)}^{l}=\sum_{j=0}^{m}a_{j}g^{j},$$

where k,l,m are integers. This function was denoted as $V_{\vec{a}}\left(k,l,m;\xi \right)$ where:

- $\vec{a}=\left(a_{0},a_{1},\cdots ,a_{m}\right)$;
- *k*: order of derivative of *g*;
- *l*: degree of derivative in the defining ODE;
- *m*: highest degree of the polynomial of *g* in the defining ODE.

The function *V* has very interesting properties. Its particular cases are the trigonometric, hyperbolic, elliptic functions of Jacobi, etc. The polynomial non-linearity in the the defining Equation (18) makes the function *V* very convenient for use in the SEsM. For more information about the use of the function *V* in the SEsM, see Appendix B.

Below, we will illustrate the use of the function $V_{\vec{a}}\left(1,2,m;\xi \right)$, which is solution of the following equation:

(19) $${\left(\frac{dg}{d\xi }\right)}^{2}=\sum_{j=0}^{m}a_{j}g^{j}$$

We note that (17) is a specific case of (19), and then the solution of (15) can be written as follows:

(20) $$\begin{array}{ccc} F(\xi ) & = & V_{{\vec{a}}_{1}}\left(1,2,5;\xi \right);\\ {\vec{a}}_{1} & = & \left(\frac{2e}{9(\alpha^{2}a-\beta^{2}b)},\frac{-2(d-3c)}{9(\alpha^{2}a-\beta^{2}b)},\frac{-2e}{3(\alpha^{2}a-\beta^{2}b)},\frac{2(d-7c)}{9(\alpha^{2}a-\beta^{2}b)},\frac{4e}{9(\alpha^{2}a-\beta^{2}b)},\frac{8c}{9(\alpha^{2}a-\beta^{2}b)}\right). \end{array}$$

Let c=d=0. Then, (15) becomes:

(21) $$au_{xx}-bu_{tt}=\frac{2}{3}e\sinh\left(\frac{2u}{3}\right)$$

The corresponding equation for *F* is:

(22) $$F_{\xi }^{2}=\frac{4e}{9(\alpha^{2}a-\beta^{2}b)}F^{4}-\frac{2e}{3(\alpha^{2}a-\beta^{2}b)}F^{2}+\frac{2e}{9(\alpha^{2}a-\beta^{2}b)}$$

Equation (21) has the following solution:

(23) $$\begin{array}{ccc} F(\xi ) & = & V_{{\vec{a}}_{2}}\left(\xi ;1,2,4\right);\\ {\vec{a}}_{2} & = & \left(\frac{2e}{9(\alpha^{2}a-\beta^{2}b)},0,\frac{-2e}{3(\alpha^{2}a-\beta^{2}b)},\frac{4e}{9(\alpha^{2}a-\beta^{2}b)}\right). \end{array}$$

We note that (22) can be written as:

(24) $$F_{\xi }^{2}=2sF^{4}-3sF^{2}+s,\;\;\;s=\frac{2e}{9(\alpha^{2}a-\beta^{2}b)}.$$

This can be further transformed to:

(25) $$F_{\xi }^{2}=s\left(1-F^{2}\right)\left(1-2F^{2}\right).$$

Equation (25) is not an equation for any of the three main Jacobi elliptic functions. The equations for the three main Jacobi elliptic functions are (0≤k≤1 is the modulus of the corresponding Jacobi elliptic function. $k^{\prime 2}=1-k^{2}$):

- F=sn(x;k): $F_{\xi }^{2}=\left(1-F^{2}\right)\left(1-k^{2}F^{2}\right)$;
- F=cn(x;k): $F_{\xi }^{2}=\left(1-F^{2}\right)\left(k^{\prime 2}+k^{2}F^{2}\right)$;
- F=dn(x;k): $F_{\xi }^{2}=\left(1-F^{2}\right)\left(F^{2}-k^{\prime 2}\right)$;

Let us now obtain another solution of (17). At Step (2.) of the SEsM, we have to relate the composite function *F* to a function *G*, which is a function of a more simple equation. The more simple equation will be of the kind (19), namely:

(26) $$G_{\xi }=kG^{4}+mG^{2}+o,$$

where k,m,o are parameters. The substitution of (26) in (17) leads to an equation for the form of the composite function. The form of the composite function is determined by the requirement that all of the coefficients of the resulting polynomial of *G* must have at least two terms. This requirement leads to a balance equation. This equation determines the maximum power of the polynomial of *G* in the relationship for the composite function *F*. The result of this operation for the simple Equation (26) is as follows:

(27) $$F\left(G\right)=pG^{2}+qG+r$$

The substitution of (26) and (27) in (17) leads to the following system of non-linear algebraic equations which connect the parameters of the solved equation and the parameters of the selected solution:

(28) $$\begin{array}{ccc} p^{2}\left(k^{2}-2cBp^{3}\right) & = & 0\\ p^{2}\left(-eBp^{2}-10cBp^{2}+2mk\right) & = & 0\\ 2p^{2}\left[2\left(m^{2}+2ok\right)-pB\left(d-7c\right)-8eBrp-40cBr^{2}p\right] & = & 0\\ 2p^{2}\left[4om-3\left(d-7c\right)Br+3eB-12eBr^{2}-40cBr^{3}\right] & = & 0\\ 2p[2po^{2}-8eBr^{3}-20cBr^{4}-3\left(d-7c\right)Br^{2}+6eBr+\left(d-3c\right)B] & = & 0\\ 2B[-e-4cr^{5}+\left(d-3c\right)r-\left(d-7c\right)r^{3}+3er^{2}-2er^{4}] & = & 0. \end{array}$$

Above $B={\left\{9\left[\alpha^{2}a-\beta^{2}b\right]\right\}}^{-1}$. One solution of the system (28) is:

(29) $$\begin{array}{ccc} p & = & 0\\ r & = & \frac{1}{12}\frac{T_{1}}{T_{2}}\\ T_{1} & = & [36edc+108ec^{2}-8e^{3}+12[12cd^{3}-108c^{2}d^{2}-3d^{2}e^{2}+324c^{3}d+126cde^{2}-\\ & & 324c^{4}-27c^{2}e^{2}-24e^{4}c{{]}^{1/2}]}^{2/3}-12cd+36c^{2}+4e^{2}-\\ & & [36edc+108ec^{2}-8e^{3}+12[12cd^{3}-108c^{2}d^{2}-3d^{2}e^{2}+324c^{3}d+126cde^{2}-\\ & & 324c^{4}-27c^{2}e^{2}-24e^{4}c{{]}^{1/2}]}^{1/3}\\ T_{2} & = & c[36edc+108ec^{2}-8e^{3}+12[12cd^{3}-108c^{2}d^{2}-3d^{2}e^{2}+324c^{3}d+126cde^{2}-\\ & & 324c^{4}-27c^{2}e^{2}-24e^{4}c{{]}^{1/2}]}^{1/3} \end{array}$$

Thus, we have obtained the following result. We obtain a solution of (17). This solution is a composite function containing the solution of Equation (26). The solution (26) can be written in terms of the *V* function:

(30) $$\begin{array}{ccc} G & = & V_{{\vec{a}}_{3}}\left(\xi ;1,1,8\right);\\ {\vec{a}}_{3} & = & \left(o^{2},0,2mo,0,2ko+m^{2},0,2km,0,k^{2}\right). \end{array}$$

Then, from (23):

$$F\left(G\right)=qV_{{\vec{a}}_{3}}\left(\xi ;1,1,8\right)+r.$$

The solution of (17) is as follows:

(31) $$\begin{array}{ccc} u(x,t) & = & 3\cosh^{-1}[(qV_{{\vec{a}}_{3}}\left(\xi ;1,1,8\right)+\frac{1}{12}[[36edc+108ec^{2}-8e^{3}+12[12cd^{3}-108c^{2}d^{2}-\\ & & 3d^{2}e^{2}+324c^{3}d+126cde^{2}-324c^{4}-27c^{2}e^{2}-24e^{4}c{{]}^{1/2}]}^{2/3}-12cd+36c^{2}+4e^{2}-\\ & & [36edc+108ec^{2}-8e^{3}+12[12cd^{3}-108c^{2}d^{2}-3d^{2}e^{2}+324c^{3}d+126cde^{2}-\\ & & 324c^{4}-27c^{2}e^{2}-24e^{4}{c]}^{1/2}{]}^{1/3}]/[c[36edc+108ec^{2}-8e^{3}+12[12cd^{3}-108c^{2}d^{2}-\\ & & 3d^{2}e^{2}+324c^{3}d+126cde^{2}-324c^{4}-27c^{2}e^{2}-24e^{4}c{{]}^{1/2}]}^{1/3}])]. \end{array}$$

The above shows that the function *V* can be very useful for use in the methodology of the SEsM. We have demonstrated that there are cases when solutions of the solved non-linear differential equation exist, but they cannot be constructed by elementary functions or by the known special functions. In these cases, the solutions can be constructed by appropriate V-functions. In addition, the V-function contains as specific cases many well-known functions. Because of all above, we emphasize this special function and we will use it in our future work.

## 5. Concluding Remarks

This article is devoted to a discussion of the following question: How can the methodology called the Simple Equations Method (SEsM) lead to exact analytical solutions of non-linear differential equations containing non-polynomial non-linearity? We follow a classic idea: to find appropriate transformation which converts the non-polynomial non-linearity to more treatable kind of non-linearity. This more treatable kind of non-linearity is the polynomial non-linearity, and the SEsM can successfully deal with such kind of non-linearity. We discuss the necessary properties of the above appropriate transformation. Several transformations which have these properties are listed. Two illustrative examples are presented. The first example shows the procedure of the application of the SEsM and leads to kink and anti-kink traveling wave solutions of the solved non-linear differential equation. The second example illustrates the application of an useful function which is solution of an ordinary differential equation with polynomial non-linearity. This function can be used in many cases when the solution of the solved equation cannot be constructed by elementary functions. 

## Appendix A. Faa di Bruno Relationship for Derivatives of a Composite Function

The composite functions and their derivatives play important roles in the SEsM. Because of this, we provide information about the Faa di Bruno relationship for the derivatives of the composite functions. Let us consider the function $h(x_{1},\cdots ,x_{d})$. It is a function of *d* independent variables $x_{1},\cdots ,x_{d}$. The function *h* is a composite function of *m* other functions $g_{1}^{\left(1\right)},\cdots ,g^{\left(m\right)}$

(A1) $$h\left(x_{1},\cdots ,x_{d}\right)=f\left[g^{\left(1\right)}\left(x_{1},\cdots ,x_{d}\right),\cdots ,g^{\left(m\right)}\left(x_{1},\cdots ,x_{d}\right)\right].$$

We use the following notations:

- $\vec{\nu }=\left(\nu_{1},\cdots ,\nu_{d}\right)$ is a *d*-dimensional index containing the integer non-negative numbers $\nu_{1},\cdots ,\nu_{d}$;
- $\vec{z}=\left(z_{1},\cdots ,z_{d}\right)$ is a *d*-dimensional object containing the real numbers $z_{1},\cdots ,z_{d}$;
- $|\vec{\nu }|=\sum_{i=1}^{d}\nu_{i}$ is the sum of the elements of the *d*-dimensional index $\vec{\nu }$;
- $\vec{\nu }!=\prod_{i=1}^{d}\nu_{i}!$ is the factorial of the multi-component index $\vec{\nu }$;
- ${\vec{z}}^{\vec{\nu }}=\prod_{i=1}^{d}z_{i}^{\nu_{i}}$ is the $\vec{\nu }$-th power of the multi-component variable $\vec{z}$;
- $D_{\vec{x}}^{\vec{\nu }}=\frac{\partial^{|\vec{\nu }|}}{\partial x_{1}^{\nu_{1}}\cdots \partial x_{d}^{\nu_{d}}}$, $|\vec{\nu }|>0$ is the $\vec{\nu }$-th derivative with respect to the multi-component variable $\vec{x}$. We note that in this notation $D_{\vec{x}}^{\vec{0}}$ is the identity operator;
- $||\vec{z}||=\max_{1\leq i\leq d}|z_{i}|$ is the maximum value component of the multi-component variable $\vec{z}$;
- For the *d*-dimensional index $\vec{l}=\left(l_{1},\cdots ,l_{d}\right)$ ($l_{1},\cdots ,l_{d}$ are integers), we have $\vec{l}\leq \vec{\nu }$ when $l_{i}\leq \nu_{i},i=1,\cdots ,d$. Then we define
  ν→l→=∏i=1dνili=ν→!l→!(ν→−l→)!.
- Ordering of vector indices. For two vector indices, $\vec{\mu }=\left(\mu_{1},\cdots ,\mu_{d}\right)$ and $\vec{\nu }=\left(\nu_{1},\cdots ,\nu_{d}\right)$, we have $\vec{\mu }≺\vec{\nu }$ when one of the following holds:
  **(a.)**$|\vec{\mu }|<|\vec{\nu }|$;
  **(b.)**$|\vec{\mu }|=|\vec{\nu }|$ and $\mu_{1}<\nu_{1}$;
  **(c.)**$|\vec{\mu }|=|\vec{\nu }|$, $\mu_{1}=\nu_{1}$, ⋯$\mu_{k}=\nu_{k}$ and $\mu_{k+1}<\nu_{k+1}$ for some 1≤k<d.

In addition, we need the following notation:

$$h_{\left(\vec{\nu }\right)}=D_{\vec{x}}^{\vec{\nu }}h;\;\;\;f_{\left(\vec{\lambda }\right)}=D_{\vec{y}}^{\vec{\lambda }}f;\;\;\;g_{\left(\vec{\mu }\right)}^{\left(i\right)}=D_{\vec{x}}^{\vec{\mu }}g^{\left(i\right)};\;\;\;{\vec{g}}_{\left(\vec{\mu }\right)}=\left(g_{\left(\vec{\mu }\right)}^{\left(1\right)},\cdots ,g_{\left(\vec{\mu }\right)}^{\left(m\right)}\right).$$

Then, The Faa di Bruno relationship for the composite derivative of a function containing functions of many variables is as follows [112]:

(A2) $$h_{\left(\vec{\nu }\right)}=\sum_{1\leq |\vec{\lambda }|\leq n}f_{\left(\vec{\lambda }\right)}\sum_{s=1}^{n}\sum_{p_{s}\left(\vec{\nu },\vec{\lambda }\right)}\left(\vec{\nu }!\right)\prod_{j=1}^{s}\frac{{\left[{\vec{g}}_{\left({\vec{l}}_{j}\right)}\right]}^{{\vec{k}}_{j}}}{\left({\vec{k}}_{j}!\right){\left[{\vec{l}}_{j}!\right]}^{|{\vec{k}}_{j}|}}.$$

In (A2), $n=|\vec{\nu }|$. Moreover:

$$p_{s}\left(\vec{\nu },\vec{\lambda }\right)=\left\{{\vec{k}}_{1},\cdots ,{\vec{k}}_{s};{\vec{l}}_{1},\cdots ,{\vec{l}}_{s}\right\},\;\;\;|{\vec{k}}_{i}|>0,$$

and

$$0≺{\vec{l}}_{1}\cdots ≺{\vec{l}}_{s},\;\;\;\sum_{i=1}^{s}{\vec{k}}_{i}=\vec{\lambda },\;\;\;\sum_{i=1}^{s}|{\vec{k}}_{i}|{\vec{l}}_{i}=\vec{\nu }.$$

For case of 1 function of 1 variable h=f[g(x)], the Faa di Bruno formula can be written as follows:

(A3) $$h_{\left(n\right)}=\sum_{k=1}^{n}f_{\left(k\right)}\sum_{p(k,n)}n!\prod_{i=1}^{n}\frac{g_{\left(i\right)}^{\lambda_{i}}}{\left(\lambda_{i}!\right){\left(i!\right)}^{\lambda_{i}}}.$$

In (A3):

- $h_{\left(n\right)}=\frac{d^{n}h}{dx^{n}}$ is the *n*-th derivative of the function *h*.
- $f_{\left(k\right)}=\frac{d^{k}f}{dg^{k}}$ is the *k*-th derivative of the function *f*.
- $g_{\left(i\right)}=\frac{d^{i}g}{dx^{i}}$ is the *i*-th derivative of the function *g*.
- $p\left(n,k\right)=\left\{\lambda_{1},\lambda_{2},\cdots ,\lambda_{n}\right\}$: set of numbers such that
  $$\sum_{i=1}^{n}\lambda_{i}=k;\sum_{i=1}^{n}i\lambda_{i}=n.$$

## Appendix B. Several Results Relevant for Applications of the SEsM in the Main Text

Here, we mention a theorem (for details about other related propositions and their proofs, see [102,113]). The theorem is connected to the case of the application of the SEsM when the composite function is constructed by a function of a single variable and this function of a single variable satisfies differential equation containing polynomial non-linearity. Let us consider a non-linear partial differential equation with non-linearity which are polynomials of the unknown function h(x,t) and its derivatives. We search for a solution of the following kind:

h(x,t)=h(ξ);ξ=μx+νt,

where μ and ν are parameters. The basis of our search will be a solution g(ξ) of a certain simple equation. Then:

(A4) h=f[g(ξ)]

*h* from Equation (A4) is a composite function. We assume that *f* is a polynomial of *g*. Then:

(A5) $$f=\sum_{r=0}^{q}b_{r}g^{r}.$$

We use the following simple equation:

(A6) $$g_{\left(k\right)}^{l}={\left(\frac{d^{k}g}{d\xi^{k}}\right)}^{l}=\sum_{j=0}^{m}a_{j}g^{j}.$$

This is the Equation (18) from the main text above. In (A6), k,l,m are integers. We remember that the solution of (A6) defines the function $V_{a_{0},a_{1},\cdots ,a_{m}}(\xi ;k,l,m$) where:

**(a.)***k*: order of derivative of *g*;
**(b.)***l*: degree of derivative in the defining ODE;
**(c.)***m*: highest degree of the polynomial of *g* in the defining ODE.

The trigonometric, hyperbolic, elliptic functions of Jacobi, etc., are specific cases of the function *V*. Below, we note a theorem in which the function $V_{a_{0},a_{1},\cdots ,a_{m}}\left(\xi ;1,2,m\right)$ participates. This function is solution of the following simple equation:

(A7) $$g_{\left(1\right)}^{2}={\left(\frac{dg}{d\xi }\right)}^{2}=\sum_{j=0}^{m}a_{j}g^{j}.$$

The theorem is as follows [102]:

**Theorem** **A1.**
*If $g_{\left(1\right)}^{2}$ is given by Equation (A7) and f is a polynomial of g given by Equation (A5), then for h[f(g)], the following relationship holds:*

$$h_{\left(n\right)}=K_{n}\left(q,m\right)\left(g\right)+g_{\left(1\right)}Z_{n}\left(q,m\right)\left(g\right)$$

*where $K_{n}\left(q,m\right)\left(g\right)$ and $Z_{n}\left(q,m\right)\left(g\right)$ are polynomials of the function g(ξ).*

This theorem allows us to calculate fast the derivatives of composite functions of interest for SEsM. The polynomials $K_{n}\left(q,m\right)\left(g\right)$ and $Z_{n}\left(q,m\right)\left(g\right)$ can be calculated as follows:

(A8) $$\begin{array}{ccc} K_{0} & = & \sum_{r=0}^{q}b_{r}g^{r}\\ Z_{0} & = & 0 \end{array}$$

Then, starting from (A8), we obtain:

(A9) $$\begin{array}{ccc} K_{n+1} & = & \frac{Z_{n}}{2}\sum_{j=0}^{m}ja_{j}g^{j-1}+\frac{dZ_{n}}{dg}\sum_{j=0}^{m}a_{j}g^{j}\\ Z_{n+1} & = & \frac{dK_{n}}{dg}. \end{array}$$

We note that the equations of Bernoulli and Riccati are specific cases of the following simple equation:

(A10) $$g_{\left(1\right)}=\sum_{j=0}^{n}c_{j}g^{j}.$$

In (A10), *n* and $c_{j}$ are constant parameters. The equation of kind (A10) occurs in the Example 2 in the main text. Equation (A10) is a specific case of (A7). This can be easily seen as follows. The idea is that (A7) contains all cases of (A10). From (A10) we obtain:

(A11) $$g_{\left(1\right)}^{2}=\left(\sum_{i=0}^{n}c_{i}g^{i}\right)\left(\sum_{j=0}^{n}c_{j}g^{j}\right)=\sum_{i=0}^{n}\sum_{j=0}^{n}c_{i}c_{j}g^{i+j}=\sum_{k=0}^{2n}a_{k}g^{k}.$$

In (A11), $a_{k}$ are appropriate combinations of the coefficients $c_{i}$. Equation (A11) is of the kind (A7). Then, (A7) contains all possible relationships of the kind (A10). However, (A7) contains more than this. For an example, (A7) contains the following case:

(A12) $$g_{\left(1\right)}^{2}=a_{0}+a_{1}g.$$

(A12) cannot be reduced to relationship of the kind (A10). Then, (A10) is a specific case of (A7).

For the case when the simple equation has the specific form (A10), we have to calculate a single kind of polynomial $L_{n}$. In other words, when the simple equation is of the kind (A10), $h_{\left(n\right)}$ is a polynomial of *g*: $h_{\left(n\right)}=L_{n}\left(g\right)$. $L_{n}$ can be calculated as follows. We start from:

$$L_{0}=\sum_{r=0}^{q}b_{r}g^{r}.$$

Then we use the recurrence relationship:

(A13) $$L_{i+1}=\frac{dL_{i}}{dg}\sum_{j=0}^{m}c_{j}g^{j}.$$

The derivatives $h_{\left(1\right)}$, $h_{\left(2\right)}$, $h_{\left(3\right)}$, $h_{\left(4\right)}$, $h_{\left(5\right)}$ are much used in the model non-linear partial differential equations. Below, we calculate the polynomials $K_{n}$ and $Z_{n}$ connected to these derivatives. We start from:

(A14) $$\begin{array}{ccc} K_{0} & = & \sum_{r=0}^{q}b_{r}g^{r}\\ Z_{0} & = & 0 \end{array}$$

From Equation (A9), we obtain:

(A15) $$K_{1}=0;\;\;Z_{1}=\sum_{r=0}^{q}rb_{r}g^{r-1}$$

Then:

(A16) $$\begin{array}{ccc} K_{2} & = & \sum_{r=0}^{q}\sum_{j=0}^{m}\left[\frac{1}{2}jr+r\left(r-1\right)\right]a_{j}b_{r}g^{j+r-2}\\ Z_{2} & = & 0. \end{array}$$

(A17) $$\begin{array}{ccc} K_{3} & = & 0;\\ Z_{3} & = & \sum_{r=0}^{q}\sum_{j=0}^{m}\left[\frac{1}{2}jr+r\left(r-1\right)\right]\left(j+r-2\right)a_{j}b_{r}g^{j+r-3} \end{array}$$

(A18) $$\begin{array}{ccc} K_{4} & = & \sum_{r=0}^{q}\sum_{j=0}^{m}\sum_{u=0}^{m}\left[\left(\frac{1}{2}jr+r\left(r-1\right)\right)\left(j+r-2\right)\left(\frac{1}{2}u+j+r-3\right)\right]a_{j}b_{r}a_{u}g^{j+r+u-4}\\ Z_{4} & = & 0. \end{array}$$

(A19) $$\begin{array}{ccc} K_{5} & = & 0;\\ Z_{5} & = & \sum_{r=0}^{q}\sum_{j=0}^{m}\sum_{u=0}^{m}\left[\left(\frac{1}{2}jr+r\left(r-1\right)\right)\left(j+r-2\right)\left(\frac{1}{2}u+j+r-3\right)\right](j\;+\\ & & r+u-4)a_{j}b_{r}a_{u}g^{j+r+u-5} \end{array}$$

We can also calculate several of the polynomials $L_{i}$ for the case (A10) We start from:

(A20) $$L_{0}=\sum_{r=0}^{q}b_{r}g^{r}.$$

The application of the recurrence relationship (A13) leads to the following relationships for $L_{1}$, $L_{2}$, ⋯:

(A21) $$L_{1}=\sum_{r=0}^{q}\sum_{j=0}^{m}b_{r}rc_{j}g^{r+j-1}.$$

(A22) $$\begin{array}{c} L_{2}=\sum_{r=0}^{q}\sum_{j=0}^{m}\sum_{k=0}^{m}b_{r}r\left(r+j-1\right)c_{j}c_{k}g^{r+j+k-2}. \end{array}$$

(A23) $$\begin{array}{c} L_{3}=\sum_{r=0}^{q}\sum_{j=0}^{m}\sum_{k=0}^{m}\sum_{l=0}^{m}b_{r}r\left(r+j-1\right)\left(r+j+k-2\right)c_{j}c_{k}c_{l}g^{r+j+k+l-3}. \end{array}$$

(A24) $$\begin{array}{c} L_{4}=\sum_{r=0}^{q}\sum_{j=0}^{m}\sum_{k=0}^{m}\sum_{l=0}^{m}\sum_{n=0}^{m}b_{r}r\left(r+j-1\right)\left(r+j+k-2\right)\left(r+j+k+l-3\right)c_{j}c_{k}c_{l}c_{n}\times \\ g^{r+j+k+l+n-4}. \end{array}$$

(A25) $$\begin{array}{c} L_{5}=\sum_{r=0}^{q}\sum_{j=0}^{m}\sum_{k=0}^{m}\sum_{l=0}^{m}\sum_{n=0}^{m}\sum_{p=0}^{m}b_{r}r\left(r+j-1\right)\left(r+j+k-2\right)\left(r+j+k+l-3\right)\times \\ \left(r+j+k+l+n-4\right)c_{j}c_{k}c_{l}c_{n}c_{p}g^{r+j+k+l+n+p-5}. \end{array}$$
